# Cellular responses to ionising radiation of AT heterozygotes: differences between missense and truncating mutation carriers

**DOI:** 10.1038/sj.bjc.6601549

**Published:** 2004-02-17

**Authors:** M Fernet, N Moullan, A Lauge, D Stoppa-Lyonnet, J Hall

**Affiliations:** 1 DNA Repair Group, International Agency for Research on Cancer, 150 cours Albert Thomas, 69372 Lyon cedex 08, France; 2 Service de Génétique, Institut Curie, 26 rue d'Ulm, 75248 Paris cedex 05, France

**Keywords:** ataxia telangiectasia, heterozygosity, ionising radiation, ATM, mutations

## Abstract

It has been estimated that approximately 1% of the general population are ataxia telangiectasia (AT) mutated (*ATM*) heterozygotes. The ATM protein plays a central role in DNA-damage response pathways; however, the functional consequences of the presence of either heterozygous truncating or missense mutations on *ATM* expression and the ionising radiation (IR)-induced cellular phenotype remain to be fully determined. To investigate this relationship, the *ATM* mRNA and protein levels and several cellular end points were characterised in 14 AT heterozygote (AT het) lymphoblastoid cell lines, compared to normal and AT homozygote lines. The AT het cell lines displayed a wide range of IR-induced responses: despite lower average levels of *ATM* mRNA and protein expression compared to normal cells, 13 out of 14 were capable of phosphorylating the ATM substrates p53-ser15 and Chk2, leading to a normal cell cycle progression after irradiation. However, cell survival was lower than in the normal cell lines. The presence of a missense compared to a truncating mutation was associated with lower cell survival after exposure to 2 Gy irradiation (*P*=0.005), and a higher level of *ATM* mRNA expression (*P*=0.047). Our results underline the difficulty in establishing a reliable test for determining *ATM* heterozygosity.

Ataxia telangiectasia (AT) is a rare autosomal recessive multisystemic disorder with neurological, cutaneous and immunological abnormalities. This disease is associated with an elevated risk of malignancy, primarily leukaemias and lymphomas, and a high radiosensitivity. The gene defective in this syndrome, *ATM* (AT mutated), encodes a nuclear 350 kDa phosphoprotein containing a carboxy terminus phosphatidylinositol 3′-kinase (PI-3′ kinase) catalytic domain shared by members of a superfamily of large eukaryotic proteins involved in intracellular signalling, DNA damage-induced cell cycle checkpoints, DNA repair and recombination (reviewed in Khanna *et al*, 2001). After exposure of cells to ionising radiation (IR) or radiomimetic drugs, ATM's kinase activity increases several-fold, although the protein level remains unchanged. In the DNA-damage response pathway, ATM acts upstream of p53 to induce cell cycle arrest at the G_1_/S and G_2_/M boundaries and a slowing of the S phase (Hoekstra, 1997). At least part of this activation mechanism involves phosphorylation of p53 on serine-15 that may contribute to the increased half-life of p53 by facilitating its dissociation from MDM2, a protein that promotes p53 proteolysis (Nakagawa *et al*, 1999). Signalling by ATM also involves interactions with and phosphorylation of the mitotic checkpoints Chk1 and Chk2 (Chen *et al*, 1999; Matsuoka *et al*, 2000). Cells from AT homozygotes show a reduced induction of p53 levels after exposure to IR compared with normal cells, and an altered cell cycle progression with an accumulation in the G_2_ phase of the cell cycle postirradiation and radioresistant DNA synthesis occurring. Loss of ATM function is also associated with increased genomic instability that contributes to the increased incidence of cancers seen in this syndrome (reviewed in Shiloh, 2003).

A wide spectrum of *ATM* mutations is found in AT patients. Most are compound heterozygous and, in some populations, there is a strong founder effect (Concannon and Gatti, 1997). About 70% of the *ATM* mutations identified in AT patients are truncating mutations (*ATM*^*trunc*^), with about 30% being missense mutations and small in-frame deletions/insertions (*ATM*^*mis*^) (Gilad *et al*, 1996; Stankovic *et al*, 1998). In many such studies, only the protein truncation test has been used to identify mutations, inevitably creating a bias in the mutations reported; thus, the percentage of truncating mutations could be an overestimate. Truncating mutations act effectively as null mutations, as they result in an unstable protein that is present in very small amounts within a cell. In contrast, missense mutations lead to the expression of a more stable albeit mutant form of the ATM protein, as has been described for the few homozygotes carriers of missense mutations that have been studied (Angele(c) *et al* (2003) and references therein).

Heterozygous carriers of the *ATM* gene (AT hets) have been estimated to comprise 0.36–1% of the general population. These individuals show none of the severe clinical symptoms seen in AT patients, but have a higher risk of developing several forms of cancer, particularly breast cancer (BC) in women and an earlier age at death from ischaemic heart disease than noncarriers (Su and Swift, 2000). On the basis of the increased risk of BC and the heterozygote frequency in the general population, it has been estimated that about 5% of BC patients could be *ATM* mutation carriers. Since the *ATM* gene was cloned, many efforts have been made to evaluate the public health implications of this observation by screening cohorts of BC patients for *ATM* mutations. The first published studies produced conflicting results, and did not reveal the magnitude of involvement of the *ATM* gene in sporadic BC that would have been expected from the increased risk found in the AT family studies and the frequency of AT heterozygotes in the population. To resolve this paradox, it has been hypothesised that there are two populations of AT carriers, one group with a truncating allele coupled with a normal allele (AT hets^trunc^) and a second group with a missense mutation coupled with a normal allele (AT hets^mis^), and that the latter group might be more prone to cancer development (McConville *et al*, 1996; Gatti *et al*, 1999; Meyn, 1999). Indeed, there is some experimental evidence that carrying an *ATM* missense mutation could lead to a dominant-negative phenotype in human cell lines (Chenevix-Trench *et al*, 2002; Scott *et al*, 2002), with competition between the mutant and the wild-type forms of the ATM protein.

The establishment of *ATM* heterozygosity is essential for the estimation of cancer risks and genetic counselling. For instance, intermediate sensitivity to IR has been documented in cell lines from AT heterozygotes using the measurement of cell survival or chromosome damage induction as end points (Chen *et al*, 1978; Rosin and Ochs, 1986; Shiloh *et al*, 1986; Scott *et al*, 1996; Tchirkov *et al*, 1997; Neubauer *et al*, 2002). However, many such studies have characterised only cell lines carrying truncating mutations, and some variability in the cellular responses has been noted between different cell lines (Nagasawa *et al*, 1985; Shiloh *et al*, 1989; Pandita and Hittelman, 1994; Djuzenova *et al*, 1999). In order to establish which methods, other than mutation screening, might provide reliable end points for determining *ATM* heterozygosity, we have compared the cellular phenotype before and after exposure to IR of AT heterozygote cell lines with that seen in cell lines carrying a wild type or homozygous mutated *ATM* gene. To assess whether *ATM*^*mis*^ and *ATM*^*trunc*^ mutations have distinct effects on ATM protein function, this comparison was made using three heterozygote cell lines carrying missense and 11 carrying truncating mutations. The end points examined were the constitutive levels of *ATM* mRNA and protein expression and the cell cycle delay in G_2_ phase, the phosphorylation of Chk2 and serine 15 of p53 and the cell survival after exposure to IR.

## MATERIALS AND METHODS

### Cell lines

Lymphoblastoid cell lines (LCLs), established by Epstein–Barr virus infection of ficoll-purified lymphocytes, were used throughout this study. The seven normal LCLs (IARC 2139, 2145, 2093, 1326, 1663, 1104 and 2209) and the six AT LCLs (IARC AT3, AT6, AT8, AT11, AT13 and AT14) were obtained from Dr Gilbert Lenoir (Institute Gustave Roussy, Villejuif, France). All the AT LCLs carried *ATM* mutations resulting in a truncated ATM protein (Gilad *et al*, 1996; Hacia *et al*, 1998; Ange(c)le *et al*, 2003). It should be noted that the AT lines IARC AT13 and AT14 were established from sibling pairs and carry the same homozygous truncating mutation. The *ATM* gene status of the normal cell lines has been previously determined using the restriction endonuclease fingerprinting technique, and all lines carried a wild-type *ATM* gene (unpublished data). The 14 AT heterozygote LCLs used in this study were all established from the parents of AT children. The *ATM* gene status in these lines was determined using the FAMA technique (Verpy *et al*, 1994). All carried either a truncating or a missense mutation (Table 1

**Table 1:** Characteristics of AT heterozygote cell lines

			**Cell survival**			**Cell**	**ATM**	**ATM**		
**Cell line**	**Mutation typePosition (amino-acid change)**	**Mutation localisation**	**1 Gy**	**2 Gy**	**4 Gy**	**cycle G2/G1**	**protein level**	**mRNA level**	**p53-ser15**	**Chk2**
1–17 178	Truncating – 381delA (ter aa1280)	Exon 7	AT	AT	AT	N	Int	AT	N	N
1–19 320	Truncating – 3403del174 (NP-58aa^a^)	Exon 26	Int	Int	AT	N	Int	Int	N	N
1–21 954	Truncating – 3576del170 (ter aa1196)	Exon 27	Int	Int	AT	N	N	Int	N	N
1–17 179	Truncating – 3711del5 (ter aa1243)	Exon 27	N	N	AT	N	N	AT	N	N
1–17 814	Truncating – 4661insA (ter aa1560)	Exon 33	AT	Int	AT	N	N	N	N	N
10–572	Truncating – C6004T (gln202stop)	Exon 42	N	Int	AT	Int	Int	Int	Int	N
1–17 180	Truncating – C7327T (arg2443stop)	Exon 52	AT	Int	Int	N	Int	Int	N	N
1–17 815	Truncating – 7789del139 (ter aa2599)	Exon 55	AT	AT	AT	N	Int	Int	Int	N
1–21 953	Truncating – G7913A (trp2638stop)	Exon 55	AT	AT	AT	N	N	Int	N	N
1–17 052	Truncating – 8030delA (ter aa2681)	Exon 57	AT	AT	AT	N	N	AT	N	N
10–573	Truncating – C8140T (gln2714stop)	Exon 57	N	N	Int	N	Int	AT	N	N
	All Truncating		Int	Int	AT	N	Int	Int	N	N
										
1–15 308	Missense – TG7875GG (asp-ala2652glu-pro)	Exon 55	AT	AT	AT	N	N	N	Int	N
1–17 053	Missense – T8489G (val2830gly)	Exon 60	AT	AT	AT	N	Int	N	Int	N
1–25 065	Missense – T8479G (phe2827ile)	Exon 60	Int	AT	AT	N	N	Int	N	N
	All Missense		AT	AT	AT	N	N	Int	N	N
										
All			Int	Int	AT	N	Int	Int	N	N

a NP – native protein lacking 58 amino acids encoded by exon 26. ter – protein termination. ‘N’ and ‘AT’ mean that the results obtained for the AT het line were not statistically significantly different from that obtained for the normal or AT LCLs, respectively. ‘Int’ means that this result was significantly different from those of both the normal and AT LCLs.

). The cell line 1–21 953 was found to be carrying a missense sequence alteration G544C and a truncating mutation trp2368stop. This missense alteration has not been previously described as a polymorphism in the *ATM* mutation database (http://www.benaroyaresearch.or g/bri_investigators/atm.htm). It has been reported as a rare variant (allele frequency 0.015) by Izatt *et al* (1999), and found in the heterozygous state in four out of 65 individuals of North African origin (Hall, unpublished data), and thus was considered as an *ATM* polymorphism in this present study.

LCLs were routinely maintained at 37°C, in a humidified incubator with 5% CO_2_, in exponential growth by dilution twice a week to 5 × 10^5^ cells ml^−1^ in RPMI1640 medium (Gibco, Invitrogen Corporation, Cergy-Pontoise, France) supplemented with 10% heat-inactivated foetal calf serum (Integro b.v, Zaandan, Holland) and penicillin and streptomycin (100 *μ*g ml^−1^, Gibco).

### ATM protein expression

A whole-cell protein extract was prepared, as previously described, from lymphoblastoid cells plated at 5 × 10^5^ cells, the day before extraction (Jongmans *et al*, 1998). 50 *μ*g of this protein extract was separated on a 6% biphasic SDS–polyacrylamide gel, transferred overnight at 30 V onto a PVDF membrane (Roche Diagnostics) using the Bio-Rad Trans-Blot Cell (Bio-Rad, Marnes la Coquette, France) and immunoblotted using an ATM antibody (AHP392, Serotec, 1/1000 dilution) and a ku80 antibody (AHP317, Serotec, 1/40 000 dilution). After a 2 h incubation with the primary antibodies, the membranes were probed with peroxidase-conjugated goat anti-rabbit IgG (DAKO, Denmark) and developed using a chemiluminescence procedure (Amersham, Piscataway, NJ, USA). The expression levels of each protein were determined by densitometry, after correction for protein loading using Ku80 as an internal standard (ATM/Ku80 ratio). Measurements were made at least twice using two independently prepared protein extracts.

### Analysis of ATM mRNA levels

#### RNA isolation and cDNA synthesis

Total RNA was isolated from 0.5 × 10^7^ cells plated at 5 × 10^5^ cells ml^−1^ the day before extraction, using the RNazol extraction method (Invitrogen). Reverse transcription was carried out on 5 *μ*g of total RNA in a 20-*μ*l reaction volume containing 1 × first-strand buffer, 200 U of Superscript II RNase H-reverse transcriptase, 40 U of RNase inhibitor (Promega, Charbonnieres, France), 10 mM DTT (Invitrogen), 125 mM of each dNTP and 0.5 *μ*g of oligo(dT)_15_ (Promega) for 1 h at 42°C. The resulting cDNA were used for real-time amplification reactions.

#### Quantitative real-time PCR

The mRNA levels of the *ATM* gene were measured following generation of the corresponding cDNA by real-time PCR based on TaqMan chemistry, and quantitated using an ABI PRISM 7900 HT sequence detector system (Applied Biosystems, Les Ulis, France). The cDNA levels of an internal control gene, the *Actin* gene, were measured and used to normalise the *ATM* cDNA levels. Oligonucleotide primers and Taqman probes for *ATM* were designed from the Genbank databases using Primer Express (Applied Biosystems). The probes for human *ATM* and *Actin* used in the PCR reactions were located in two adjacent exons of each gene to avoid amplification of any genomic DNA contaminating the cDNA.

The *ATM* forward primer was 5′-GAGCAGAGTCTTGCCCTGAGTATT-3′ and the reverse primer was 5′-TTGCCACAAACCCTCAGACA-3′.

The *Actin* forward primer was 5′-CTGGCACCCAGCACAATG-3′ and the reverse primer was 5′-GCCGATCCACACGGAGTACT-3′.

The Taqman probe for *ATM* was 5′-CTGTGCAGCGAACAATCCCAGCCT-3′ and had a fluorescent reporter dye (FAM) covalently linked to its 5′ end. The Taqman probe for *Actin* was 5′-TCAAGATCATTGCTCCTCCTGAGCGC-3′ and had a fluorescent reporter dye (TET) covalently linked to its 5′. The nonfluorescent quencher dye BHQ1 was linked to the 3′ end of each probe. The reaction mixture contained 0.2 *μ*l of cDNA template, 50 nM of *Actin* primers, 2 *μ*M of *ATM* primers, 100 nM of TaqMan *ATM* probe, 200 nM of TaqMan *Actin* probe and 1 × TaqMan universal master mix (Applied Biosystems). Amplification and detection of specific products was carried out in an ABI PRISM 7900 HT sequence detection system (Applied Biosystems) using an amplification protocol consisting of an initial denaturing and enzyme activation at 95°C for 10 min, followed by 35 cycles at 94°C for 15 s and 62°C for 1 min. For each sample, three independent RNA extractions have been analysed, with each corresponding cDNA analysed six times on the same plate.

The threshold cycle (Ct), defined as the cycle where the amplification of the PCR product enters the exponential phase, was determined for each gene by plotting the fluorescence level *vs* the cycle number on a logarithmic scale. The relative expression level of the *ATM* gene in the different cell lines was then estimated using a semiquantitative method, by calculating the DCt value, defined as the difference in the Ct value for the target (*ATM*) and the reference gene (*Actin*). The results are expressed as the ratio 100/DCt for ease of interpretation.

### Cell survival

The radiation survival of the LCLs was measured by their relative growth at 72 h postirradiation (Gilad *et al*, 1996). The cells were seeded at 2 × 10^5^ cells ml^−1^ the day before irradiation and cell survival was assessed 3 days after exposure to 0, 1, 2 and 4 Gy from a ^137^Cs source, at a dose rate of about 5 Gy min^−1^, by counting the number of living cells using trypan blue exclusion. The number of viable cells in the nonirradiated culture 72 h postirradiation was considered as 100% cell survival. Experiments were performed at least twice on each cell line with at least one normal and one AT LCL being analysed in parallel.

### p53 and Chk2 phosphorylation

Measurements of p53-ser15 and Chk2 phosphorylation were made in total protein extracts prepared from control or irradiated (5 Gy) LCLs (10^7^ cells plated at 5 × 10^5^ cells ml^−1^ the day before). Briefly, cells were collected from untreated or treated cells 2 and 4 h after treatment, washed twice in PBS and lysed in TGN buffer (50 mM Tris-HCl pH 7.5, 150 mM NaCl, 1% Tween 20, 0.2% Nonidet-P40, 0.2 mg ml^−1^ pefabloc^R^ (Boehringer, Roche Diagnostics, Grenoble, France), 1 mM sodium fluoride, 1 mM sodium orthovanadate, 10 *μ*g ml^−1^ aprotinin, 2 *μ*g ml^−1^ pepstatin A and 5 *μ*g ml^−1^ leupeptin for 30 min on ice. The supernatant was collected after centrifugation for 15 min at 15 000 r.p.m. (4°C), and its protein concentration determined using the BIO-RAD protein assay. 50 *μ*g of this protein extract was separated on a 10% biphasic SDS–polyacrylamide gel and transferred overnight onto a PVDF membrane, as described above. The p53-ser15 phosphorylation was measured using a specific p53-phosphoserine 15 antibody (#9284, Cell Signaling Technology, 1/1000 dilution), and the Chk2 phosphorylation was detected by the mobility shift of the protein using a specific Chk2 antibody (477, Abcam, 1/1500 dilution). The expression levels of each protein were determined by densitometry, after correction for protein loading using Ku80 as an internal standard, as described above. Measurements were made at least twice from a minimum of two independently prepared protein extracts.

### Cell cycle analysis

7.5 × 10^6^ cells (plated at 5 × 10^5^ cells ml^−1^) were plated into two flasks and left to grow overnight. One flask was subsequently used as a control and the second treated with 5 Gy of IR. At 0, 24 and 48 h postirradiation, an aliquot of the control and treated cells were collected, washed in PBS and treated with propidium iodide using the CycleTest™ Plus Kit (Becton Dickinson, Pont-de-Claix, France), according to the manufacturer's instructions. The DNA content was analysed by flow cytometry (FACSCalibur, Becton Dickinson), using the CellQuest software; 10 000 events were analysed for each sample. The percentage of cells in each phase of the cell cycle was determined using the ModFit programme. Experiments were performed at least in duplicate on each cell line. The cell cycle defect was investigated by calculation of the ratio (% of cells in G_2_ phase)/(% of cells in G_1_ phase), representative of the delay in G_2_ occurring after exposure to IR (Lavin *et al*, 1992).

### Statistical methods

The differences in the basal levels of *ATM* mRNA and ATM protein, cell survival and the induction of the phosphorylation of p53 in response to DNA damage between the LCLs established from the normal donors, the AT homozygote patients and the AT heterozygotes have been tested by the analysis of the variance (ANOVA). For the intergroup comparison, significant heterogeneity in the variance was assessed by the Bartlett test, and, if necessary, transformations of the variables were applied: arc sine of the square root for cell survival after 1 and 2 Gy, and logarithm for ATM protein level and p53 phosphorylation. For some comparisons, it was impossible to stabilise the variance, so a Mann–Whitney *U*-test was used (*ATM* mRNA level, G_2_/G_1_ ratio and cell survival after 4 Gy). All the statistical computations were performed using the STATISTICA software (version 5.97, Statsoft).

## RESULTS

### ATM protein and mRNA levels in normal, AT heterozygote and AT cells

**Figure 1:**
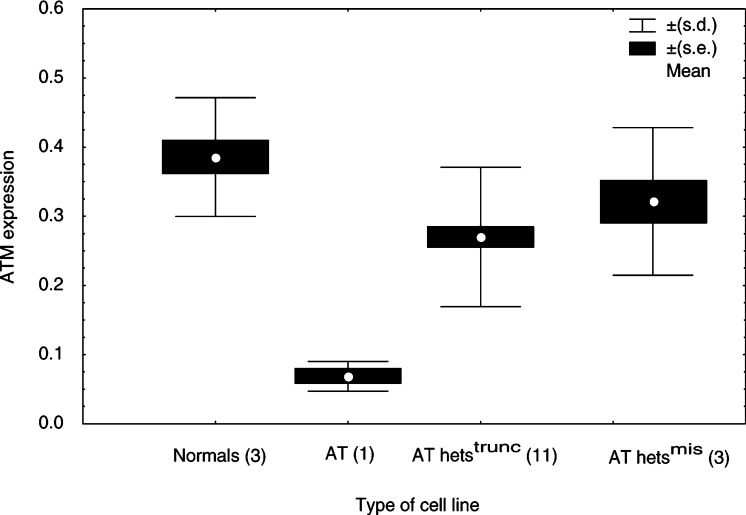
ATM protein levels in the normal (IARC 2093, 2145, 2139), AT (AT11), AT hets^trunc^ and AT hets^mis^ LCLs examined. The level of ATM was calculated from the densitometric analysis of Western blot films, after correction for protein loading using Ku80 as a marker. The average intensity±s.d. was determined for each group of cell types, with at least two measurements made for each line (*n*=number of lines in each group).

The mean values of ATM protein levels for the 14 AT het cell lines, three carrying missense mutations and 11 truncating mutations, in comparison to the three normal cell lines analysed, are presented in Figure 1

. The AT cell line (AT11) carrying two heterozygous truncating mutations, with a very low level of ATM protein that is a characteristic of cells from many AT patients, is included as a negative control. On an individual basis, half of the AT het cell lines examined showed a 20–30% lower expression of ATM protein (Table 1) compared to the normal cell lines. As a group, the AT het LCLs expressed significantly less ATM protein than the normal cell lines (*P*=0.002), but more than AT11 (*P*<0.001). There were no statistical differences in the protein levels between the three AT hets^mis^ LCLs studied, compared to the 11 AT hets^trunc^ LCLs (*P*=0.110) studied. However, the AT hets^trunc^ as a group had a statistically lower ATM protein level than the normal cell lines (*P*<0.001), while the group of AT hets^mis^ were indistinguishable from the normal LCLs in terms of ATM protein expression (*P*=0.102).

**Figure 2:**
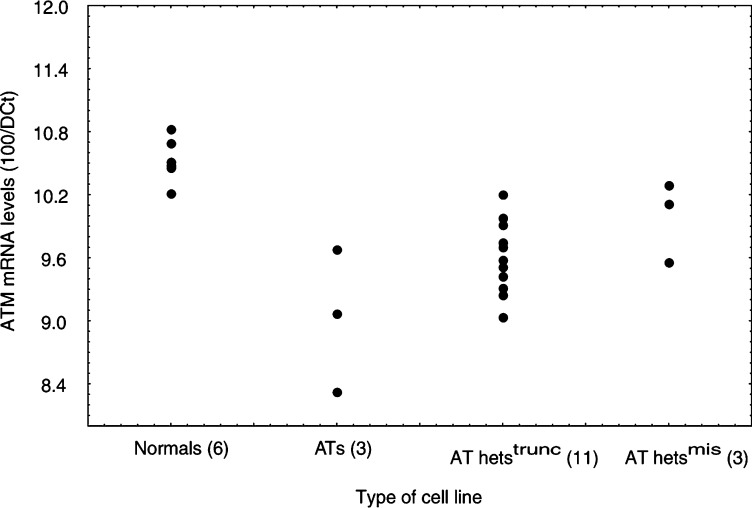
ATM-mRNA level in the six normal, three AT (AT6, AT11, AT14), 11 AT hets^trunc^ and three AT hets^mis^ LCLs. The mRNA level, expressed as the ratio 100/DCT, was obtained by TaqMan analysis and correction for RNA content using *β*-actin. Data points represent the mean value from at least three experiments for each of the cell lines studied (*n*=number of lines in each group), except for AT6 and AT14, which were assayed twice.

The mean values of a ratio proportional to the DCt values, representative of the *ATM* mRNA level, for the six normal, three AT and all the AT het cell lines are presented in Figure 2

. As a group, the AT het cell lines exhibited an intermediate mRNA level lower than that of the normal (*P*<0.001) and higher than that seen in the AT cell lines (*P*<0.001), with some variation between the different lines being seen. The AT hets^mis^ as a group had lower *ATM* mRNA levels than the normal cell lines (*P*=0.002), and higher levels than the AT (*P*<0.001) and AT hets^trunc^ (*P*=0.047) cell lines. The mRNA levels in the AT hets^trunc^ cell lines were also intermediate between the normal (*P*<0.001) and the AT lines (*P*<0.001).

### Cell survival

**Figure 3:**
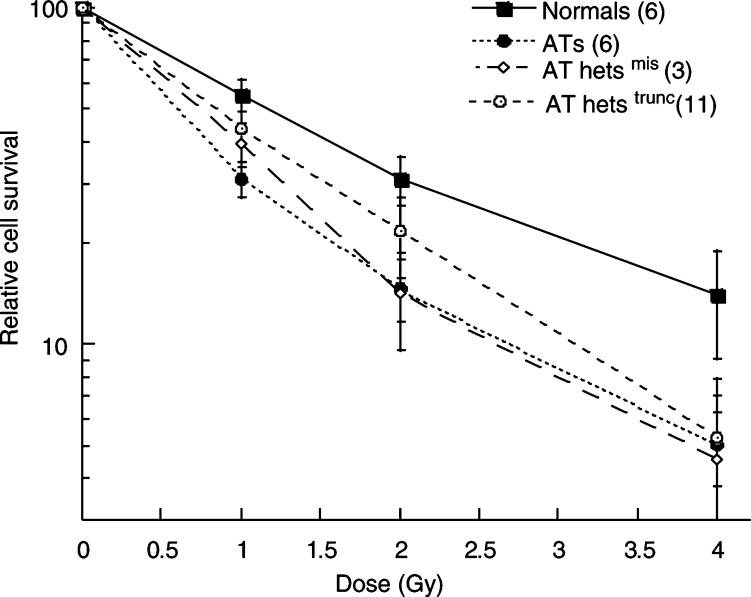
Radiation cell survival curves. The viable cells were counted by trypan blue exclusion 3 days after exposure to gamma radiation. Mean values±s.d. for each group of cell lines were established based on the results from at least two independent experiments on each line.

The relative cell survival of the cell lines studied after exposure to 1, 2 and 4 Gy is shown in Figure 3

. Statistical analysis of the results was done for each dose separately. The cell survival of the six normal and the six AT cell lines was of the same order, as previously found, under identical experimental conditions (Ange(c)le *et al*, 2003). Considerable variation was seen between the different AT het cell lines but, as a group, they had a cell survival level which was intermediate and distinguishable between that seen in the normal (*P*<0.001) and AT (*P*<0.001) LCLs after exposure to 1 and 2 Gy. It was, however, indistinguishable from that of the AT LCLs after exposure to 4 Gy (*P*=0.604).

Differences were noted in the cell survival profile between the two groups of AT het LCLs studied. The group carrying a missense mutation had a lower average cell survival, which was distinguishable from that of the LCLs carrying truncating mutations after exposure to 2 Gy (*P*=0.005), but not after exposure to 1 Gy (*P*=0.271). The survival of the AT hets^mis^ was indistinguishable from that of the AT LCLS after exposure to 1 and 2 Gy (*P*=0.072 and 0.803, respectively), while the AT hets^trunc^ group showed a higher survival at both doses (*P*<0.001 for 1 and 2 Gy). After exposure to 4 Gy, the two groups of AT hets were as radiosensitive as the group of AT LCLs (*P*=0.509 and 0.755 for AT hets^trunc^ and AT hets^mis^, respectively).

### Phosphorylation of Chk2 and p53-serine 15

**Figure 4:**
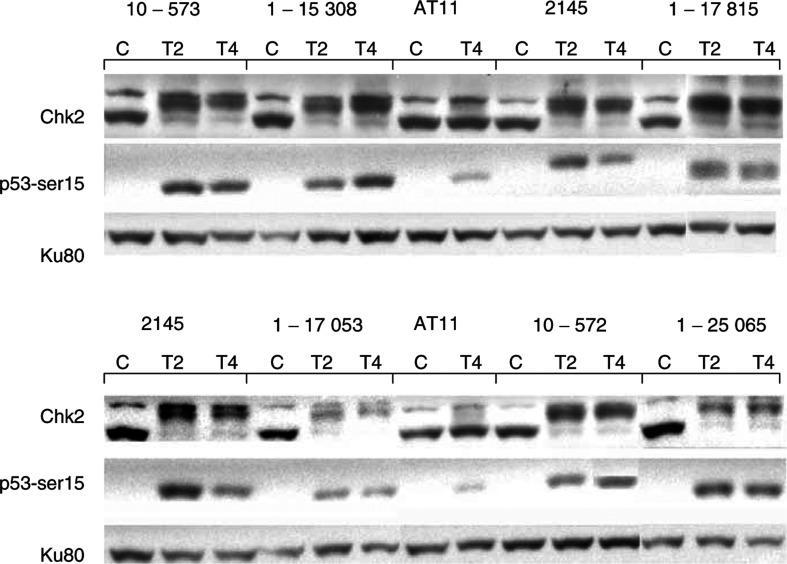
Radiation-induced Chk2 and p53-ser15 phosphorylation. Protein extracts were prepared from the normal (IARC 2145), AT (AT11) and AT heterozygote (10–573, 1–15 308, 1–17 815, 1–17 053, 10–572 and 1–25 065) LCLs 2 and 4 h after exposure to 5 Gy, and from untreated cells. Ku80 immunodetection was performed on each gel to correct for variations in protein loading. Phosphorylation of Chk2 was assessed by the mobility shift of the protein band, while the intensity of the p53-ser15 bands was analysed by densitometry.

**Figure 5:**
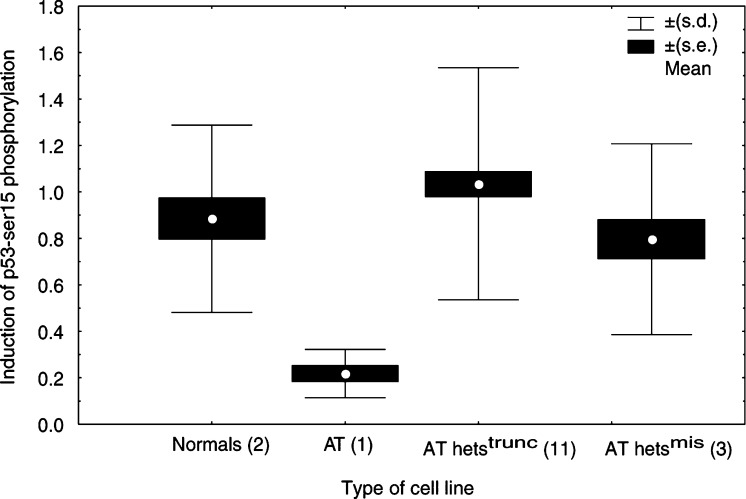
Quantification of the p53-ser15 phosphorylation, presented as the p53-ser15/ku80 ratio to correct for variation in protein loading, 2 and 4 h after exposure to 5 Gy IR. The average ratio±s.d. was calculated for each group of cell lines, based on at least two independent experiments for each line (*n*=number of lines).

The phosphorylation of the two ATM-target proteins Chk2 and p53-ser15 was studied 2 and 4 h after a 5 Gy irradiation by Western blot analysis (Figure 4

). The AT het cell lines were compared with two normal and an AT LCLs, representative of each cell type. As expected, the AT11 LCL exhibited a reduced phosphorylation of p53-ser15 and no shift in the position of Chk2, 2 or 4 h after exposure to IR, while the normal cell lines showed a maximum p53-ser15 phosphorylation 2 h after treatment and a clear shift in the mobility of Chk2. The average levels of the p53-ser15 induction for the four cell types are shown in Figure 5

. No differences were noted between the group of AT hets^mis^ or AT hets^trunc^ and the normal LCLs (*P*=0.355 and 0.279, respectively). However, on an individual cell line basis of the 14 AT hets LCLs studied, a delayed and reduced p53-ser15 phosphorylation was noted in four lines (10-572, 1-17815, 1-15308 and 1-17053). All the AT het cell lines showed a Chk2 bandshift after exposure to IR, as seen in the normal cell lines.

### Cell cycle progression

**Figure 6:**
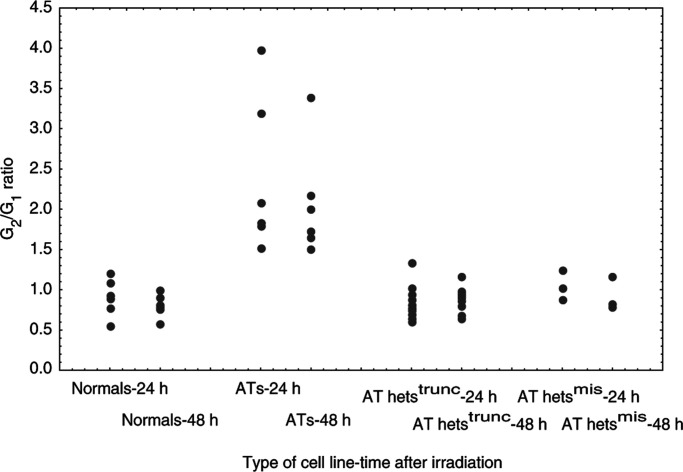
Cell cycle delay in G_2_ phase. This delay was estimated in six normal, six AT and the 14 AT het cell lines, by calculation of the G_2_/G_1_ ratio, 24 and 48 h after exposure to 5 Gy. Data points represent the average value for each cell line, based on at least two independent experiments.

The radiation-induced arrest in the G_2_ phase of the cell cycle was assessed by calculating the ratio between the percentage of cells in G_2_ and the percentage of cells in G_1_, 24 and 48 h after irradiation (Figure 6

). All the statistical analyses were made by taking into account the results at 24 and 48 h after irradiation. As previously noted, the six AT cell lines showed a greater cell cycle delay in G_2_, reflected in a higher G_2_/G_1_ ratio, compared to the six normal cell lines (*P*<0.001) (Ange(c)le *et al*, 2003). As a group, the 14 AT het cell lines presented a G_2_/G_1_ ratio similar to that of the normal cell lines (*P*=0.382), with no differences being noted depending on the type of mutation being carried (*P*=0.894 and 0.494 for AT hets^trunc^ and AT hets^mis^, respectively). We, however, noted that two AT het LCLs, both carrying a truncating *ATM* mutation, showed a higher G_2_/G_1_ ratio than the normal LCLs (AT het cell line 10-572, *P*=0.025 and 1-17815, *P*=0.056).

## DISCUSSION

It has been postulated and supported by limited experimental evidence that the presence of *ATM* missense mutations, in contrast to *ATM* truncating mutations, may result in a different cellular phenotype after exposure to IR and alter the risk of developing cancers in heterozygote carriers (Stankovic *et al*, 1998; Gatti *et al*, 1999; Chenevix-Trench *et al*, 2002; Scott *et al*, 2002). We have investigated the constitutive levels of expression of the *ATM* gene and the *in vitro* cellular phenotype after exposure to IR in 14 heterozygote cell lines carrying different mutations located throughout the *ATM* gene, to assess whether mutation-type specific differences could be detected and whether such end points could provide a reliable assay for the identification of *ATM* carriers. A wide range of responses was noted, extending from that seen in normal cell lines to the extreme reactions seen in homozygote carriers of *ATM* mutations, with the profile of responses of each line varying in its severity depending on the end point being measured and the treatment dose (Table 1).

As a group, the AT heterozygotes had a reduced level of *ATM* mRNA expression, which was reflected in a lower average constitutive level of protein expression, compared to the normal LCLs studied. The assessment of the gene expression phenotype has been proposed as a possible means of identifying *ATM* heterozygotes (Watts *et al*, 2002), and these results would lend support to this proposal. However, it should be noted that some variation between the different LCLs was noted and no direct relationship between the ATM protein and mRNA levels was seen in all cell lines (Jongmans *et al*, 1997; Becker-Catania *et al*, 2000; Sun *et al*, 2002). In this study, the three AT heterozygote cell lines carrying a missense mutation had higher levels of mRNA levels, as measured by a semiquantitative PCR-based technique compared to the lines carrying truncating mutations, reflecting the observations in *ATM* homozygote lines. However, differences in ATM protein levels between the two groups of AT hets was not so clearcut, as has been previously reported (Shigeta *et al*, 1999; Becker-Catania *et al*, 2000; Delia *et al*, 2000). It should be noted that, under the experimental conditions used, no truncated forms of the ATM protein were detected in any of the heterozygote lines examined. It cannot be excluded that differences in expression may also exist between the two *ATM* alleles, as has been found to be the case in XP-C heterozygotes where the expression of the normal allele predominated (Chavanne *et al*, 2000).

Enhanced cell killing in response to treatment with IR is one characteristic of all *ATM* homozygote cells, but the level of this radiosensitivity in *ATM* heterozygote cells appears more variable. Many authors have reported a level of cell survival intermediate between that seen in normal and *ATM* homozygote cells, but, in other studies, some overlap or no statistically significant differences between normal and AT het cell types have been detected (Chen *et al*, 1978; Kinsella *et al*, 1982; Nagasawa *et al*, 1985; Weeks *et al*, 1991; West *et al*, 1995; Shigeta *et al*, 1999; Sun *et al*, 2002). Many of these studies have used a fibroblast-cloning assay, which requires the trypsinising and replating of treated cells, which may lead to additional cell death, particularly in radiation-sensitive cell types. Using a cell proliferation assay, which avoids the manipulation of the treated cells, our results indicated a greater radiosensitivity for the AT heterozygote cells as a group, compared with the normal cells. Some overlap between the results obtained from the AT heterozygote and homozygote cells lines was noted, in particular after exposure to 4 Gy, suggesting that such high doses are not the ideal experimental conditions to distinguish these two cell types. When the distinction between the carriers of missense or truncating mutations was made, we noted that the cell lines carrying missense mutations were more radiosensitive than those carrying truncating mutations after treatment. This observation was based on the overlap of their survival with the AT cell lines at lower doses, which was not seen for the AT het^trunc^ lines, and the fact that the two groups could be distinguished after exposure to 2 Gy. These findings suggest that the ATM protein containing a missense mutation could act in a dominant-negative manner influencing cell survival.

To evaluate the kinase activity of the ATM protein in the heterozygote cells, the *in vivo* phosphorylation of the p53-ser15 and Chk2 was assessed after exposure to IR. The AT cell line studied exhibited an approximately five-fold reduced p53-ser15 phosphorylation compared to the two normal cell lines under the experimental conditions used. This p53-ser15 phosphorylation is probably due to the kinase activity of ATR (ATM-related), which is also able to carry out this specific p53 phosphorylation (Matsuoka *et al*, 2000). However, no Chk2 phoshorylation, as assessed by the alteration of its mobility on the SDS–PAGE gels, was noted in this homozygote line. In contrast, under these same experimental conditions, Chk2 phosphorylation was detected in all the 14 AT heterozygote cell lines, and 10 of the 14 cell lines showed a normal level of p53-ser15 phosphorylation. A possible explanation of this result is that a second kinase activity capable of carrying out these reactions is present in these heterozygote lines at a higher level than seen in normal cell lines, such as increased levels of ATR. It should be noted that these two end points were assessed on the same Western blots, so that these apparently substrate-specific differences could not be explained by variability between protein extracts, but reflect the *in vivo* pattern of phosphorylation that had occurred following exposure to IR. No mutation-type (truncating *vs* missense) specific differences in the capacity to phos-phorylate these ATM-target proteins were noted. Some variation between ATM kinase activity has been reported in the limited number of heterozygote lines previously studied (Delia *et al*, 2000; Scott *et al*, 2002), suggesting that these end points are not suitable for evaluating the presence of *ATM* heterozygosity with certitude.

Alterations in the activation of p53, Chk2 and other proteins lead to disturbances in cell cycle progression after irradiation. Under such conditions, most AT cell lines fail to delay at the G_1_/S and G_2_/M checkpoints, progressing through the cell cycle to accumulate and die in G_2_/M. Thus, the G_2_/G_1_ ratio can be used to estimate the accumulation of cells in G_2_/M after exposure to IR. We found that the AT het cell lines as a group exhibited a delay in G_2_ similar to that seen in the normal cell lines after such treatment. An intermediate G_2_ delay has been reported previously in some AT het cell lines between that observed in normal and AT cell lines, but overlaps in the response between these three cell types have also been reported (Lavin *et al*, 1992; Chen *et al*, 1994; Naeim *et al*, 1994). One explanation for these differences in the cell cycle profile could be the radiation treatment dose used. In this present study, the cell cycle progression was examined after an exposure to 5 Gy, while other studies have used lower treatment doses (2 or 3 Gy). It can be reasoned that, even if the pathways controlling the cell cycle progression are not fully operational in AT heterozygote cells, after exposure to higher doses of IR with greater levels of DNA damage being formed, the activation of cell cycle regulation could occur, perhaps through the intervention of other DNA damage-activated kinases, leading to an apparently normal response. The lack of a defect in cell cycle progression in the majority of the AT het LCLs examined after exposure to IR is consistent with the normal activation of p53 and Chk2 observed, two proteins strongly involved in cell cycle checkpoint control. The one AT het line which had a statistically lower G_2_/G_1_ ratio also showed a lower p53-ser15 phosphorylation after treatment with IR, but a normal Chk2 response underlining the complex interplay of the proteins controlling the cell cycle response to DNA damage. It would appear in spite of a reduced ATM protein level in some *ATM* carriers that this endogenous protein is sufficient to ensure the functionality of the signalling pathways leading to cell cycle control after exposure to IR, but not to ensure normal levels of cell survival.

The variation between the different AT heterozygote lines studied, especially in terms of *ATM* mRNA and protein levels and cell survival, is large with some cell lines being indistinguishable from normal cell lines, while others have a cellular phenotype similar to that seen in AT homozygote lines. One explanation for this interindividual variability might be the location of the different *ATM* mutations within the gene, which may affect ATM protein expression and/or its activity. Morgan *et al* (1997) demonstrated that the expression of an ATM fragment encoding the leucine zipper domain can act in a dominant-negative manner influencing cell survival, but not p53 induction nor cell cycle checkpoints, while expression of the PI-3 kinase domain is sufficient to ensure ATM kinase activity (Morgan *et al*, 1997). The mutations carried by the AT heterozygote cell lines included in this present study were located throughout the *ATM* gene, and all the truncating mutations were located upstream of the conserved PI-3 kinase domain (codon 60/61). Thus, if a stable truncated protein was expressed, it would contain the leucine zipper domain and could potentially act in a dominant-negative manner, influencing cell survival, as has been demonstrated by Morgan *et al* (1997). However, its presence would not be expected to impact on the kinase activity, which would originate solely from the protein expressed from the wild-type allele. It is formally possible that alternative start codons in the *ATM* coding region may exist downstream of the premature termination codons carried in the *ATM* heterozygotes. Translation initiating at such codons might produce an ATM fragment corresponding to the PI-3 kinase domain that could retain kinase activity, as has been shown by Morgan *et al* (1997). Such an alternative mode of translation has been found in Nijmegen breakage syndrome patients carrying the common founder mutation 657del5 allele, a truncating mutation that causes premature termination at codon 219. In these individuals, an NBS protein lacking the native N-terminus is produced by internal translation initiation within the *NBS1* mRNA using an open-reading frame generated by the 657del5 frameshift (Maser *et al*, 2001).

This study, characterising several aspects of the DNA damage response in 14 AT heterozygote cell lines, highlights their diversity and the difficulties associated with finding a reliable test for the detection of *ATM* carriers in the general population. Based on the results obtained from the experimental approaches used here, none of the end points alone would allow the assessment of *ATM* heterozygosity with a 100% accuracy. As a group, the cell lines with such a genetic profile have reduced *ATM* mRNA and protein levels, and a lower cell survival following exposure to IR, but on an individual basis some considerable variation is seen, with some AT heterozygote lines resembling lines carrying a wild-type *ATM* gene. The presence of a missense mutation compared to a truncating mutation is associated with a higher level of *ATM* mRNA expression, but a lower cell survival, after exposure to IR. The determination of the gene expression profile in AT heterozygotes, in particular genes implicated in cell survival, where the clearest differences were observed experimentally between the carriers and noncarriers of an altered *ATM* gene, is a promising avenue to be pursued. Watts *et al* (2002) have also recently reported that LCLs from AT heterozygotes have a specific expression phenotype based on baseline and induced changes in response to IR. One important practical consideration of any approach to identify *ATM* carriers is the need to extend the results to peripheral blood lymphocytes, which will eliminate the requirement of establishing cell lines and enable its use in a clinical setting.
